# Editorial: The essential role of multidisciplinary teams in breast cancer surgery: collaboration for superior patient outcomes

**DOI:** 10.3389/fonc.2025.1754900

**Published:** 2026-01-07

**Authors:** Raquel Diaz, Francesca Poggio, Alessandra Fozza, Irene Giannubilo, Letizia Cuniolo, Piero Fregatti

**Affiliations:** 1 Department of Surgical and Diagnostic Integrated Sciences (DISC), University of Genova, Genova, Italy; 2 Medical Oncology Unit 2, Istituto di Ricovero e Cura a Carattere Scientifico (IRCCS) Ospedale Policlinico San Martino, Genoa, Italy; 3 Department of Radiation Oncology, IRCCS Ospedale Policlinico San Martino, Genova, Italy

**Keywords:** breast cancer surgery, collaborative care, multidisciplinary team (MDT), patient-centered approach, precision medicine

## Introduction

Breast cancer remains one of the most common and complex diseases affecting women worldwide, and its management increasingly depends on the integration of multiple medical disciplines. In recent years, advances in imaging, genetics, and surgical techniques have significantly changed our approach, but the most important element has been the development of multidisciplinary teamwork. The idea that no single specialty can fully address the needs of breast cancer patients has evolved from concept to reality. Today, successful treatment is not only about the precision of surgery or the accuracy of diagnosis, but about the coordination, dialogue, and shared expertise that bring those elements together.

This Research Topic, “*The Essential Role of Multidisciplinary Teams in Breast Cancer Surgery: Collaboration for Superior Patient Outcomes*” was designed to explore how collaboration drives progress in breast cancer care. The eleven papers gathered in this Research Topic reflect the remarkable range of perspectives that define modern practice, from the operating room to the radiology suite, from pathology and oncology to psychology and rehabilitation. Together, they illustrate that true innovation arises when disciplines meet around the same patient.

## The value of collaboration

Multidisciplinary care has become the cornerstone of comprehensive breast cancer management. Team discussions allow for individualized treatment planning, the alignment of oncologic and reconstructive goals, and improved communication with patients and their families. Beyond the practical aspects, these meetings foster a shared culture of decision-making where every professional contributes their expertise to a common purpose.

The articles included in this Research Topic demonstrate this principle in different but complementary ways. Some highlight how close collaboration between radiologists and pathologists prevents misdiagnosis in rare entities, as in the reports of mucinous carcinoma in a male patient or adenoid cystic carcinoma with multiple metastases. Others, such as those describing benign lesions initially suspected as malignancies, remind us how dialogue between specialists can prevent unnecessary procedures and anxiety for patients.

Technological advances also play a growing role within multidisciplinary settings. Two studies in this Topic explore the use of artificial intelligence and radiomics in ultrasound evaluation, showing how computational models can assist clinicians in predicting therapeutic response and improving diagnostic accuracy. However, these studies also show that technological progress reaches its real potential only when embedded in multidisciplinary collaboration.

Surgical innovation is another area where collaboration proves essential. Papers addressing complex postoperative complications, such as refractory seroma, or describing multidisciplinary management of chemotherapy extravasation, provide tangible examples of how teamwork translates into better outcomes. Similarly, the study exploring combined lung biopsy and microwave ablation for suspected metastases expands the boundaries of breast surgery, integrating thoracic expertise into oncologic care.

Finally, the human dimension of multidisciplinary care is powerfully represented by the study on pain management from the ARISE project. By involving anesthesiologists, oncologists, psychologists, and rehabilitation specialists, it underscores that addressing pain is not merely a technical issue but a holistic challenge requiring shared responsibility.

Reading across all these papers, several lessons emerge. Communication itself is therapeutic: regular, structured discussion among specialists leads to earlier and more accurate decisions. Innovation thrives on integration: new technologies, from AI to image-guided surgery, succeed only when evaluated through collective expertise. True personalization of breast cancer treatment depends on teamwork, combining clinical, molecular, and psychosocial insights. And sustained education in interdisciplinary collaboration ensures that these advances endure across generations.

Taken together, this Research Topic goes beyond the technical or academic level. It captures a shift in mindset: from isolated excellence to collective intelligence, showing that progress in breast cancer surgery is built on communication and trust between disciplines.

## Conclusion

The contributions gathered in this Research Topic collectively affirm that multidisciplinary teamwork is no longer optional in breast cancer surgery: it is essential. Each article illustrates a different facet of collaboration, whether in diagnosis, operative strategy, complication management, or supportive care. Together, they provide a snapshot of a field that continues to evolve toward integration, precision, and humanity.

As Guest Editors, we are deeply grateful to the authors and reviewers who have contributed to this project. Their commitment and expertise reflect the very principles this Topic celebrates: communication, cooperation, and shared purpose. We hope this Research Topic will serve as both a scientific reference and an inspiration for future endeavors, reminding us all that in breast cancer surgery, the best outcomes are achieved when we work together across disciplines, across institutions, and always in partnership with our patients.

